# Supplementary material for: Hippopotamus are distinct from domestic livestock in their resource subsidies to and effects on aquatic ecosystems
Source: Proc Biol Sci. 2020 Apr 29;287(1926):20193000. doi: 10.1098/rspb.2019.3000 (PMC7282896; doi:10.1098/rspb.2019.3000)
Supplement: Electronic supplementary material [file rspb20193000supp1.docx]

**Electronic Supplementary Material**

**Masese, F.O., Kiplagat, M.J., González-Quijano, C.R., Subalusky, A.L., Dutton, C.L., Post, D.M. & Singer, G.A. 2020. Tables, figures, detailed methods, models and statistics from “Wild mammalian herbivores are distinct from domestic livestock in their resource subsidies and ecosystem effects”.**

**Journal**: Proceedings of the Royal Society B: Biological Sciences

**Article URL:** http://dx.doi.org/10.1098/rspb.2019.3000

**This file includes:**

Electronic supplementary material S1: Characteristics of dung in African savanna and loading rates of organic matter (dung) by cattle and hippopotamus in the Mara River, kenya

Electronic supplementary material S2: Experimental mesocosms and design and characteristics of hippo dung and cattle dung used in experiement

Electronic supplementary material S3: Dung treatment effects on nutrients and organic matter

Electronic supplementary material S4: DOM composition

Electronic supplementary material S5: Modeling metabolism

Electronic supplementary material S6: Weekly measures of Ecosystem Metabolism

1. **Electronic supplementary material S1:**
2. **Characteristics of dung for large mammalian herbivores of the African savanna**

Dung from herbivore species varies considerably in C:N:P stoichiometry because of differences in body size, quality of their diet (foraging strategy) and digestive physiology (e.g. foregut and hindgut fermenters, or for comparison ruminants and non-ruminants [1, 2]. For example, the dung of browsers has higher N concentrations than that of grazers [3], which results in differences in dung C : N : P ratios [2].

**Table S1**: Mean dung C, N, P concentrations and stoichiometry for some large mammalian herbivores of the African savanna

| **Herbivore species** | **Digestive physiology and feeding strategy*** | **C (mg g ^-1^)** | **N (mg g -1)** | **P (mg g -1)** | **C:N** | **C:P** | **N:P** | **C:N:P** | **References** |
| --- | --- | --- | --- | --- | --- | --- | --- | --- | --- |
| Bushbuck | Ruminant browser | 417.0 | 18.9 | 3.3 | 25.0 | 164.0 | 6.1 | 164:6.1:1 | [2] |
| Giraffe | Ruminant browser | 499.0 | 23.9 | 3.3 | 16.1 | {Sileshi, 2017 #211}142.0 | 9.1 | 142:9.1:1 | [2] |
| Goat | Ruminant browser | 29.7^#^ | 1.7^#^ | 0.3^#^ | 19.2 | 102.3 | 6.4 | 102:6.4:1 | [4] |
| Cattle | Ruminant grazer | 29.1^#^ | 1.3^#^ | 0.5^#^ | 23.3 | 79.2 | 3.6 | 79:3.6:1 | [4] |
| Zebu cattle | Ruminant grazer | 28.4^#^ | 11.3 | 2.2 | 30.4 | 155.2 | 5.1 | 127:5.1:1 | this study |
| Buffalo | Ruminant grazer | 348.0 | 10.9 | 2.4 | 30.5 | 153.0 | 5.3 | 153:5.3:1 | [2] |
| Hartebeest | Ruminant grazer | 403.0 | 8.6 | 3.0 | 52.9 | 153.0 | 3.6 | 153:3.6:1 | [2] |
| Reedbuck | Ruminant grazer | 389.0 | 17.1 | 3.1 | 21.9 | 103.0 | 4.8 | 103:4.8:1 | [2] |
| Waterbuck | Ruminant grazer | 379.0 | 14.5 | 2.9 | 29.1 | 145.0 | 5.3 | 145:5.3:1 | [2] |
| Wildebeest | Ruminant grazer | 358.0 | 13.5 | 3.5 | 27.3 | 119.0 | 4.8 | 119:4.8:1 | [2] |
| Zebra | Non-ruminant grazer | 414.0 | 12.2 | 3.5 | 45.7 | 213.0 | 4.0 | 213:4.0:1 | [2] |
| Hippopotamus | Non-ruminant grazer | 33.7^#^ | 9.8 | 1.3 | 34.4 | 261.4 | 7.6 | 261:7.6:1 | this study |
| Hippopotamus | Non-ruminant mixed feeder | 34.9^#^ | 1.0^#^ | 0.2^#^ | 34.9 | 222.8 | 6.3 | 223:6.3:1 | [5] |
| Elephant | Non-ruminant mixed feeder | 447.0 | 13.5 | 1.8 | 34.4 | 221.0 | 7.9 | 221:7.9:1 | [2] |

*Herbivore species were grouped per digestive physiology and feeding strategy based on [3, 6, 7].

^#^These numbers are % per weight of dry matter

1. **Loading of organic matter (dung) by cattle and hippopotamus in the Mara River, Kenya**

**Methods**: We estimated cattle loading rates of organic matter (dung) into the Mara River, Kenya inside and outside of the Maasai Mara National Reserve (MMNR), where there is an overlapping distribution of livestock and hippotamus. We used literature estimates of the daily dry matter intake (DMI) of Zebu cattle, which were determined as a fraction of their body mass (BM) [8, 9]. It has been established that DMI scales with BM in herbivores [10, 11]. We used literature values to determine DMI, which has been estimated to range from 1.9 % - 2.5 % BM for cattle on African pasture [8, 9]. Two values have been provided in the literature for BM of cattle in the Mara Basin; 180 kg [12] and 350 kg [13], so we used an average weight of 265 kg. Similarly to estimates of hippopotamus loading rates that have been determined for the Mara River [5], we used the low intake value of 1.9 % BM (equivalent to 5035 g DM) for the wet season (lasting 6 months) and the highest value of 2.5 % BM (6625 g DM) for the dry season. We assumed that the cattle population in the Mara were in metabolic equilibrium and used the following equation to estimate the mass of organic matter (OM) excreted or egested (ex/eg):

Mass of ex/eg OM = Mass Food Consumed (DMI) x % ex/eg OM

To determine the amount of OM (dry matter) excreted or egested, we used literature estimates showing cattle on a grass diet excrete or egest 57% [14]. Cattle preferentially defecate in rivers during watering or crossings [15], so we used time budgets to estimate the per cent of excretion and egestion occurring in the river. Both cattle and hippos have long mean gut retention times for particles (71 h for hippopotami and 66 h for cattle) and fluids (26 h for hippopotami and 32 h for cattle) [10, 11, 16]. Thus, we assumed constant excretion and egestion rates by cattle throughout the day, as was done in the hippopotamus study [5]. Using behavioural data collected at livestock watering points across the Mara River basin (Iteba J, unpublished data), we determined that cattle spend an average of 10 minutes in or near the river during watering and/ or crossings. To obtain the daily per-cattle rate of loading into the Mara River, we multiplied total per-cattle daily excretion and egestion rates by the fraction of time spent in the river per day.

We estimated the total loading rates to the Mara River by multiplying the per-cattle loading rate by cattle population estimates in the Mara River in 2002 [17], and compared these with the hippopotamus population in 2006 [18]. We then compared the loading of cattle and hippopotami in two areas of the Mara River where their distribution overlaps: the Mara River outside the Maasai Mara National Reserve and along the Talek River. We assumed that all cattle within either the Mara or Talek sub-catchment visited the river for watering or crossing at least once per day. Some cattle may use water pans for their water needs during certain portions of the year, which may make our loading estimates on the upper end of potential inputs.

**Results**: We estimate cattle in the Mara basin have a daily dry matter intake of 25 g DM kg^-1^ in the dry season and 19 g DM kg^-1^ in the wet season. This is in comparison to the daily dry matter intake of 4.5 g DM kg^-1^ in the wet season and 6.8 g DM kg^-1^ in the dry season for hippopotamus [5]. We estimate that an average cattle excretes or egests 10.5 g DM kg cattle^-1^day^-1^ in the wet season, and 13.8 g DM kg cattle^-1^day^-1^ in the dry season. Assuming that cattle consumption is averaged over 6 months of wet season and 6 months of dry season [5], and that they spend 10 minutes in the river per day, we estimate an average cattle loads 22.3 g DM kg cattle^-1^day^-1^ to the river. Using % dry mass estimates from cattle faeces in the field (25.7% dry mass), we calculated that 22.3 g DM equals 86.6 g faeces (wet mass), thus an average cattle (265 kg) defecates 12.5 kg faeces (wet mass) every day, and 0.0866 kg (0.69% of daily defecation) of that goes into the Mara River. In comparison, an average hippopotamus (1500 kg) defecates 17.4 kg faeces (wet mass) every day, and 8.7 kg (50%) of that goes into the Mara River [5]. Using population estimates from 2000 [12, 17], we estimated total daily loading for the cattle population in the Mara River outside the reserve (MMNR) and along the Talek River to be 2599 kg and 7364 kg faeces (wet mass), respectively (Table S1). Although the cattle population estimates of 2000 are old, a study shows that by 2016 the numbers had not changed significantly, althouh the numbers were higher between 2005 and 2010 [19]. In comparison, the total daily loading from excretion and egestion of hippopotamus population in the Mara River outside the reserve (MMNR) and along the Talek River (1,571 and 648 individuals, respectively) is estimated to be 13,668 kg and 5,638 kg faeces (wet mass), respectively, which is equivalent to a total of 4,586 kg day^-1^ DM (Table S1). Of the total organic matter loading due to cattle and hippos, cattle contribute 6-57% of inputs.

**Table S2**. Estimated loading rates of organic matter (dung) by cattle and hippopotamus in the Mara River, Kenya.

| **Hippopotamus and cattle populations and loading numbers** | **Inside Reserve** | **Outside Reserve** | **Talek River** |
| --- | --- | --- | --- |
| Hippoptamus numbers^x^ | 1,924 | 1,571 | 648 |
| Cattle numbers* | 13,350 | 30,000 | 85,000 |
| Total loading by hippopotamus population (kg day^-1^, wet wt)^y^ | 16,739 | 13,668 | 5,638 |
| Total loading by cattle population (kg day^-1^, wet wt) | 1,157 | 2,599 | 7,364 |

*Data sources- [12, 17]. Cattle numbers outside the reserve are for the Koyake Group Ranch, while numbers for the Talek represent all other Group Ranches, estimated from the conservative number of 100,000 cattle in the group ranches outside the MMNR.

^x^Hippopotamus numbers and density are from [5, 18], respectively.

^y^Estimates of hippopotamus loading rates are from [5].

1. **Electronic supplementary material S2: Characteristics of hippo dung and cattle dung and experimental mesocosms**
2. **Characteristics of cattle and hippo dung**

Macro- and micro-nutrient composition of of cattle and hippopotamus faecal samples used for the mesocosm experiment were analysed at the Leibniz Institute for Zoo and Wildlife Research, Berlin, Germany (Table S3). Before the analysis, all dried samples (60⁰C for 48h) were grounded with an IKA A 11 Basic mill (IKA-Werke GmbH & Co. KG, 79219 Staufen, Germany) to a particle size of about 1mm. For C and N, samples were weighed and loaded into tin cups and analysed on a elemental analyser (Hekatech-Elemental analyser, Thermo Finnigan). For P, samples were weighed, ashed in a muffle furnace at 550 °C, then digested before analysis on a Perkin-Elmer ICP-OES (Perkin Elmer, Ueberlingen, Germany). Crude protein was calculated as 6.25*N [20] vvvvvv. For analyses of carbohydrates (sucrose, d-glucose, d-fructose, starch) we used enzymatic tests, commercial kits from r-biopharm (R-Biopharm AG, 64297 Darmstadt, Germany) in which standard solutions were included. Additionally, a lab standard always was run in all nutrient analyses to check for reproducibility and accuracy of the tests. For mineral analysis (Ca, Mg, Fe, K) samples were microwave digested and analyzed by AAS (Atom-Absorption-Spectroscopy).

**Table S3**: Characteristics of hippo dung and cattle dung used in the mesocosms in this study.

| **Parameter** | **Hippo dung** | **Cattle dung** |
| --- | --- | --- |
| Mean particle sizes in mm* | 17.8 | 0.4 |
| Carbon (% of dry matter) | 33.71 | 28.36 |
| Nitrogen (% dry matter) | 0.98 | 1.13 |
| Protein (% dry matter) | 6.13 | 6.55 |
| Fructose (mg g^-1^) | 0.00 | 0.00 |
| Glucose (mg g^-1^) | 0.51 | 0.52 |
| Sucrose (mg g^-1^) | 0.26 | 0.47 |
| Starch (mg g^-1^) | 2.59 | 1.87 |
| Ca (mg g^-1^) | 6.15 | 8.07 |
| Fe (mg g^-1^) | 3.79 | 4.14 |
| K (mg g^-1^) | 9.62 | 10.29 |
| Mg (mg g^-1^) | 1.63 | 1.69 |
| P (mg g^-1^) | 1.29 | 2.23 |
| N (mg g^-1^) | 9.81 | 11.32 |
| C:N:P | 261.4:7.6:1.0 | 127.2:5.1:1.0 |

**From [21, 22].*

1. **Experimental set-up of mesocosms**

Mesocosms were constructed out of PVC canvas measuring 4.2 m long and 19 cm wide [23]. Water was recirculated in each mesocosm by paddlewheels affixed to a shaft that was powered by a motor, with each shaft (blocks A, B and C) handling 6 streams (Figure S1). The streams were located in an open field, and the entire array was covered with a shade cloth to yield even light distribution. Mesocosms were lined with washed gravel and filled with river water from a region upstream of most herbivore inputs. Mean (±SD) velocity and depth across channels were 0.078 ± 0.013 m s^-1^ and 7.8 ± 0.7 cm, respectively. Water levels were maintained by rainfall and additions of rainwater. The river water had the following physicochemical characteristics: total suspended materials = 1.11±0.1 mg L^-1^, temperature = 19.4±0.7 ⁰C, dissolved organic carbon = 1.62±0.5 mg L^-1^, nitrate = 1.48±0.4 mg L^-1^, soluble reactive phosphorus = 0.06±0.06 mg L^-1^, and a concentration of ammonia below detection limits (10 μg L^-1^). Background nutrient and DOC concentrations were lower than treatment level concentrations in all treatments.We had three replicates for each of 6 dung treatments in a replacement design ranging from 100% hippo dung to 100% cattle dung: H100 = 100% hippo, 0% cattle; H80 = 80% hippo, 20% cattle; H60 = 60% hippo, 40% cattle; H40 = 40% hippo, 60% cattle; H20 = 20% hippo, 80% cattle; and H0 = 0% hippo, 100% cattle.

Fresh hippo dung and cattle dung were collected from hippo paths and Maasai livestock pens, respectively. Dung from 5 different hippo paths and 3 cattle pens was thoroughly homogenized in buckets before use. We had three replicates for each of 6 dung treatments in a replacement design ranging from 100% hippo dung to 100% cattle dung with 20% increments of replacement (Figure S1). In contrast to a simpler pure hippo vs. pure cattle design, this approach allowed us to test for potential interactive effects between dung types, recognizable by non-linear responses to the dung treatment gradient. Treatments were randomly distributed among mesocosms, with a replicate of each treatment in each of the three blocks. A total of 120 g (wet weight, 1.7 g L^-1^) of dung was distributed in each mesocosm once at the beginning of the experiment in order to study ecosystem responses arising from differences in dung quality due to nutrient leaching and mineralization rates. This concentration of dung is lower than field estimates for hippo sites in the Mara river [4 g L-1, 5], but it provided a sufficient quantity to elicit ecosystem responses without creating hypoxic conditions.

To accelerate biofilm growth, mesocosms were inoculated with periphyton scraped off rocks from the Amala River. Each mesocosm was lined with 6 unglazed ceramic tiles that were used for weekly sampling of biofilms. Each week, one tile from each mesocosm was destructively sampled without replacement, and biofilm was scrubbed off into a known volume of water and filtered through pre-weighed and pre-combusted GF/F filters (Whatman International Ltd., Maidstone, England) for analysis of ash-free dry mass (AFDM).

**Figure S1**: Experimental set-up and dung used in mesocosms: (a) allocation of dung treatments in three blocks driven independently by paddle wheels, (b and c) layout and details of mesocosms, (d) hippo dung, and (e) cattle dung.

1. **Electronic supplementary material S3: Dung treatment effects on nutrients and organic matter**

Water samples for ammonium, nitrate, nitrite, and SRP were filtered on site through pre-combusted (450°C for 4 h) and pre-washed Whatman GF/F filters into acid-washed HDPE bottles, and stored at 4°C until analysis within 48 hr. For TSS and POM, water was filtered on site through pre-combusted and pre-weighed GF/F filters. Water samples for DOC concentration and composition were filtered on site through a double layer of pre-combusted Whatman GF/F filters (pore size 45 µm) followed by GF/75 filters (pore size 3 µm) into acid-washed and pre-combusted glass vials. DOC samples were then acidified with 2 N hydrochloric acid (HCl) to pH 2 and refrigerated at 4°C until analysis. TP and TN was determined following the persulfate digestion method [24]. We measured SRP, TN, TP, NO_3_^-2^ and NH_4_^+^ in water samples using standard colorimetric methods [24]. We measured DOC concentration using a Shimadzu TOC-V-CPN fitted with an inorganic C removal unit. We extracted Chl-*a* in 90% ethanol and determined concentrations spectrophotometrically [24]. We measured TSS concentration (g L^− 1^) by drying filters with the adhered sediments and subtracting the filter weight. POM in TSS was further determined gravimetrically after ashing filters at 450 °C for 4 h, re-weighing them, and subtracting the ashed weight from TSS. Biofilm biomass (AFDM) was measured similarly to POM using the filtered slurry from the scraped tiles and expressed per unit area.

1. **Dung treatment effects on nutrient concentrations**

**Figure S2.** Influence of dung treatment on (a) soluble reactive phosphorus (SRP), (b) nitrite, (c) ammonium, and (d) nitrate concentrations. Asterisks are displayed for significant linear relationships across low-high proportions of cattle dung for each sampling occasion (α ≤ 0.05). **P < 0.05, **P < 0.01, ***P < 0.001.*

**Figure S3.** Influence of time on (a) soluble reactive phosphorus (SRP), (b) nitrite, (c) ammonium, and (d) nitrate concentrations among dung treatments.

1. **Dung treatment effects on organic matter**

**Figure S4.** Influence of dung treatment on a) DOC, b) chlorophyll-a (Chl-*a*), c) ash-free dry mass (AFDM), d) total suspended solids (TSS), and e) particulate organic matter (POM) concentrations. Asterisks and model fits are displayed for significant linear relationships (α ≤ 0.05). **P < 0.05, **P < 0.01, ***P < 0.001.*

1. **Electronic supplementary material S4: DOM composition**

Animal dung releases dissolved organic matter of carbon (DOC) into water with nutrients. DOC in water contains thousands of molecules [25] that influence ecosystem processes through light attenuation and nutrient availability [26]. For instance, the optically active part of DOC, which is known as coloured dissolved organic carbon (CDOC), is several times that of chlorophyll in coastal areas [25]. Determining the composition of DOC allows the study of factors such as land use and land management practices, as they affect spatial and temporal variations in stream biogeochemistry and ecosystem functioning [26-29]. Here, we studied DOC released by leachates of cattle dung and hippo dung in order to understand and compare their properties and influence on ecosystem processes.

We characterized the optically active DOC fraction by absorbance and fluorescence analyses, which provide proxies for DOC source and/or biological availability [27, 30]. DOC absorbance spectra (250–600 nm, every 5 nm) and fluorescence excitation–emission matrices (EEMs, excitation wavelength from 250 to 600 nm, in 5 nm increments and emission range of 250–550 nm in 1.77 nm increments) were measured simultaneously on a Horiba Aqualog (Horiba Ltd, Kyoto, Japan) spectrophotometer using a 1 cm quartz cuvette and a scan speed of 12,000 nm min^-1^ with a response time of 0.01 s. MilliQ water was used as an optical blank. Naperian absorption coefficients were calculated from absorbance scans [31] and used to calculate a number of indices. A ratio of absorption coefficients *E_2_:E_3_* (a_250_:a_365_)_,_ which declines with increasing molecular size, was used to provide further information on DOC aromaticity and molecular weight [32]. The spectra slope ratio (S_R_), which is a ratio of the short wavelength slope (S_275-295_) and the long wavelength slope (S_350-400_), served as an indicator of molecular weight and photodegradation-induced shifts [32]. The DOC-standardized specific UV absorption at 254 nm (SUVA254), which is commonly used as an indicator of aromaticity [33], was computed by dividing decadal absorbance by cuvette path length (in m) and by DOC concentration (in mg C L^-1^).

EEMs were corrected for the water Raman scatter, Rayleigh–Tyndall effect and the inner filter effect [34, 35], and used to calculate three fluorescence indices: fluorescence index (FIX) [34], freshness index (β/α) [36], and humification index (HIX; unitless) [37]. The FIX provides information on DOC origin*,* distinguishing terrestrially derived DOC (FIX~1.2) from microbially derived DOC (FIX~1.9), and was calculated as the ratio of emission intensity at 450–500 nm for an excitation of 370 nm [34]. β/α indicates the proportion of recently produced DOC relative to more decomposed DOC [35, 36]. β/α values >1 indicate that DOC is primarily of autochthonous origin and values 0.6-0.8 indicate primarily allochthonous origin [38]. HIX is directly proportional to the humic content of DOC, where HIX values around 1–2 are associated with non-humified plant material and values > 10 are commonly reported for fulvic acid extracts [37, 39].

We used parallel factor analysis (PARAFAC) to decompose 349 EEMs into fluorescent components of DOC [40]. PARAFAC was conducted using DOMFluor toolbox 1.7 following Stedmon & Bro (2008) in Matlab 7.11.0 (MathWorks, Massachusetts, USA). The number of components was determined by using split half validation and assessed with random initialization fits and residual analysis [40]. We further characterized DOC using size-exclusion chromatography (SEC) [41], which separates three size fractions: humic substances (HS), high-molecular weight non-humic substances (HMWS) and low molecular weight substances (LMWS).

Fluorescence EEMs were very dissimilar and occurred over a wide range of excitation (ca. 250−450 nm) and emission (ca. 270−600 nm) wavelengths (Figures S4 and S5). The PARAFAC model consisted of seven components (referred as C1–C7) whose fluorophores were compared with literature (Table S2). The position and spectral shape for the seven components are shown in Figures S4 and S5. Four humic-like (C1, C4, C5 & C6), one reduced humic-like (C2) and two protein-like (C3 and C7) fluorescence components were identified across our dataset, with C1, C3, C5, and C6 being among the most commonly observed components in aquatic ecosystems [42]. C1 and C4 are located in the fluorescence region that usually define the ubiquitous humic-like Peaks C and A, respectively [43], and are related to high molecular weight humic substances of terrestrial origin [27]. In addition, component 4 has been shown to be resistant to photodegradation [44]. C5 was similar to peak M, and resembled components of high molecular weight, humic-like, terrestrial material [27] with increased aromatic carbon content, indicating higher plant material as a likely source [45]. C6 had both a primary excitation peak (ca 250−270 nm) and a secondary excitation peak (340−420 nm), which have been associated with large molecular size, hydrophobic compounds [46]. Protein-like C3 and C7 spectra resemble those of tryptophan and tyrosin free amino acids, respectively, and have been classified as originating from microbial DOM sources [27, 45]. C7 was also the most redshifted component in our study, resembling peak T [27].

**Figure S5**. Observed excitation and emission wavelengths for maximum fluorescence of the 7 PARAFAC components identified in our dataset.

**Figure S6**. Emission and excitation loadings of the 7 PARAFAC components.

**Table S4**: Fluorescent components of DOM as identified by parallel factor analysis (PARAFAC). Given are observed excitation and emission wavelengths for maximum fluorescence, alignment with distinct fluorescence peaks and PARAFAC components identified in previous studies, probable sources of DOC and a literature-based component description^a^.

| **PARAFAC component (this study)** | **Excitation**  **maximum**  **(nm)** | **Emission**  **maximum**  **(nm)** | **Peak name and PARAFAC component s (previous studies)** | **Probable sources*** | **Description** |
| --- | --- | --- | --- | --- | --- |
| C1 | <250, 250 | 428-444 | C^Ca,Cb,Cd^, M^Cd^, β^P^, 1^Sma^, 4^SMb^, 1^Ma^, 11^CMK^ | T, A, M | UVA humic-like component. Low molecular weight, biological activity, widespread |
| C2 | <250, 250 | 516-530 (500-550) | 4^CMK^ | T, M | Hydroquinone-like component. reduced humic-like component |
| C3 | 270-276 | 320-332 | B^Ca^, δ^P^, 8^CMK^, 6^SMa^, 7^SMb^, 5^SMB^, 7^Ma^, 6^Mb^, 4^CK^ | T, A, M | Protein- and tryptophan-like component, microbial-produced, widespread |
| C4 | <250, 250 | 436-456 | A^Ca,Cb^, A^Cb^, α^P^ | T | UVC humic-like, fulvic acid component. |
| C5 | <250, 250 | 378-382 | A^Cb^, M^Cd^, β^P^, 1^Sma^, 4^SMb^, 1^Ma^ | T, A, M | UVA humic-like component. Polycyclic aromatic, increased aromatic carbon content. |
| C6 | 256-262 (366-378) | 446-472 | A^Ca,Cd^, C^Ca,Cd^, α^P^ | T | UVC humic-like + UVA humic-like component. reduced humics, widespread. |
| C7 | 254 | 302 | B^Cb,Cd^, T^Cd^, γ^P^, 13^CMK^, 4^SMa^, 8^SMb^, 1^Ma^, 7^Mb^ | T, A, M | Protein- and tyrosine-like component. may indicate more degraded peptide material |

^a^Value in parentheses is secondary maximum. See text for discussion of probable origins. * T, terrestrial plant or soil organic matter; A, autochthonous production; M, microbial processing.

^ca^ Coble, Green [47]; ^cb^Coble [43]; ^cd^Coble, Del Castillo [48]; ^P^Parlanti, Wörz [35]; ^SMa^Stedmon and Markager [49]; ^SMb^Stedmon and Markager [44]; ^Ma^Murphy, Stedmon [50]; ^Mb^Murphy, Hambly [51]; ^CMK^Cory and McKnight [45]; ^SMB^Stedmon, Markager [52]; ^CK^Cory and Kaplan [53].

1. **Electronic supplementary material S5: Modeling metabolism**

We estimated flume-scale GPP and ER following Fuss, Behounek [54] by fitting a differential equation model [55, 56] to diel DO concentration measured at a single site [57, 58]. The model simulates temporal changes in DO concentration (*d*DO/d*t*) as the result of parameterized GPP, ER and reaeration (RF, eqn 1):

$\frac{d\mathrm{DO}}{dt}=\left( \mathrm{GPP}-ER+RF \right)\times\frac{1}{z}$ (1)

where GPP adds DO to the water by photosynthesis; ER consumes DO and RF is the gas exchange at the water–air interface. GPP (g O_2_ m^-2^ min^-1^) was modelled with light saturation (Ratkowsky, 1986; Uehlinger, König & Reichert, 2000) as:

$GPP=\frac{\mathrm{PAR}}{P_{1}+P_{2}+PAR}$ (2)

where PAR (W m^-2^) is the observed, instantaneous PAR. P1 (W min g^-1^ O_2_) is the inverse of the slope of a photosynthesis–irradiance curve at low light intensity and P2 (m2 min g^-1^ O_2_) is the inverse maximum photosynthesis rate. Daily GPP (GPP24, g O_2_ m^-2^ day^-1^) was integrated from P1, P2, the light record and the time step $\Delta$t between light measurements:

$GPP24=\sum_{t=t_{0}}^{t end} \frac{{PAR}_{t}}{P_{1}+P_{2}+{PAR}_{t}} \times\Delta t$ (3)

Since ER (g O_2_ m^-2^ min^-1^) is a strongly temperature-dependent process [59], it was modelled with the van’t Hoff–Arrhenius equation [60]:

$ER=\frac{\mathrm{ER}_{20}}{(24 \times60)} \times\theta^{(T-20)}$ (4)

where ER24_20_ (g O_2_ m^-2^ day^-1^) is the daily rate of ER standardized to 20 °C and *T* (°C) is the observed, time specific ambient stream temperature, and $\theta$ (theta) is the temperature dependance on respiration. Because different authors have used different values of $\theta$ [e.g., 61], and our modeling efforts were not successful with the commonly used value of 1.045, we decided to model this value and obtained a value of 1.1085 that we used in our model. Since diurnal variations in temperature in the mesocosms was high (mean daily range 14 °C - 26 °C), using a higher value for theta was more relevant for our analysis. Moreover, our model outputs were greatly improved. In order to investigate ER at *in situ* temperature, we translated ER24_20_ to ER24*_insitu_* (g O_2_ m^-2^ day^-1^) using recorded *in situ* temperature measurements *T* (°C) for every time interval $\Delta$t:

${ER24}_{insitu}=\sum_{t=t_{0}}^{t end} \frac{{ER}_{20}}{(24\times60)} \times{1.1085}^{{(T}_{t}-20)}\times\Delta t$ (5)

The reaeration flux RF (g O2 m^-2^ min^-1^) was computed as

$\mathrm{RF}=k \times\mathrm{DO}_{\mathrm{deficit}}$ (6)

where *k* is the temperature-dependent vertical gas exchange velocity (m min^-1^) and DO_deficit_ (g m^-3^) is the difference of the observed DO concentration (DO) to DO at 100% saturation (DO_Sat_):

$\mathrm{DO}_{\mathrm{deficit}}=\mathrm{DO}_{\mathrm{Sat}}- \mathrm{DO}$ (7)

DO_Sat_ was calculated from observed, time-specific ambient stream temperature and atmospheric pressure [62]. The vertical gas exchange velocity *k* (m min^-1^) is related to the reaeration coefficient *K* (min^-1^) by multiplication with depth (m) [57, 63]. We used a reaeration coefficient measured in 6 mesocosms (2 each for each block) by degassing water by boiling and then cooling in air-tight containers before carefully filling the mesocosms with minimal bubbling. The slope of the linear increase in DO concentration was used as an estimate of re-aeration. Temperature dependence of gas exchange was calculated according to Elmore [64] and Bott [65]:

$K_{T}=K_{20}\times{1.024}^{T-20}$ (8)

where *K_T_* and *K_20_* are reaeration coefficients at ambient stream temperature *T* and at 20 °C, respectively. For model fitting, the time derivative *d*DO/d*t* of eqn (1) was approximated by differences in $\Delta$DO/$\Delta$*t* across the observed time intervals, and a discretized time series of DO was predicted using observed, time-specific temperature and light conditions, barometric pressure and a chosen parameter set P1, P2, ER24_20_ and K_20_  [54-56]:

${{\mathrm{DO}_{t+1}=\mathrm{DO}_{t}+(\mathrm{GPP}_{t}- \mathrm{ER}}_{t}+\mathrm{RF}}_{t})\times\Delta t\times\frac{1}{z}$ (9)

DO_t+1_ (g O_2_ m^-2^) was computed from $\mathrm{DO}_{t}$ and GPP, ER and RF were computed from temperature and light conditions at the previous time point *t*. $\Delta t$, the time interval between *t* and *t* + 1, is needed to scale up the minute-specific rates accordingly and is chosen in agreement with the observed time series. Equation (9) was obtained by forward differencing or Eulerian integration of eqn (1) (Soetaert & Herman, 2009). A first observed DO measurement is used as a starting value ($\mathrm{DO}_{t0}$ ), from which all subsequent $\mathrm{DO}_{t}$ values are computed. To fit P1, P2, ER24_20_ and K_20_ to empirical data, we used eqn (9) in an inverse modelling approach that repeatedly models a DO time series with updated parameter values and minimizes the sum of squared residuals of the modelled to the observed DO time series. We estimated a reaeration coefficient (*k*) by filling 6 clean mesocosms (2 for each block) with degassed (boiled and cooled) water and then used recorded DO and temperature to model reaeration (K_20_) without GPP and ER. K_20_ was then used as a starting value to reliably model P1, P2, ER24_20_ and K_20_.

Over the last decade, temperature depencency of ER is an active topic of discussion and different authors have used different values (theta) for this dependence [61, 66, 67]. Our attempts to use a value of 1.045 [68] were unsuccessful, so we decided to use a higher modeled value of 1.1085. To arrive at this theta value (1.1085), we selected 50 days from different dung treatments and different days from among the 44 days experimental period (6 weeks) and modeled theta along with P1, P2 and ER (4-parameter model) and used a fixed reaeration coefficient (*k*) that we measured in our mesocosms. An average theta value was then obtained from successfully modeled days (see below), which we then fixed for a 3-parameter model (P1, P2 and ER modeled and *k* and theta fixed) we subsequently used to model all days of the experiment. Since ER increases with temperature, using a higher value for theta was more relevant for our data, which displayed a wide range in water temperature (mean daily range 14 °C - 26 °C).

A number of checks were done to pick the number of days that were successfully modeled and whose results were used for subsequent analyses. First, we used nlm in the metabolism FIT function to minimize the negative log-likelihood between measured and modeled DO values. Low values (< -100) of sum of squared residuals for each model were considered indicative of a successful and constrained fit. Secondly, model fits (graphs) were inspected to confirm that the modeled DO values perfectly or closely matched measured DO values (Figure S5). Finally, the modeled outputs for GPP and ER were inspected to make sure that they made sense. For instance, cases where GPP values were negative or ER values were zero or positive were discarded.

*Sensitivity analysis*

To determine the effect of using different values of theta on our estimates of GPP and ER, we performed a sensitivity analysis and re-run the model using a value of theta (1.045) that is common in the literature. By using a higher value of theta (1.1085), our model outputs were better constrained, i.e., the sum of squared residuals of the modeled to the measured DO time series obtained using a theta value of 1.1085 were lower for most streams compared with when a theta value of 1.045 was used (Figure S5). Better performance of the higher value of theta was also confirmed by the higher number of days that were successfully modeled: Of the 567 days out of 774 days (18 streams x 43 days) that had complete data, 400 days (70.5%) were successfully modeled by a theta value of 1.1085, while only 311 days (54.9%) were successfully modeled by the common theta value of 1.045.

The metabolism results obtained using the two theta were different, but trends in GPP, ER, GPP:ER ad NEP in response to dung treatment were generally similar (Figures S6 and S7). In both cases (Figures S6 and S7), GPP, GPP:ER and NEP increased with increasing proportions of cattle dung. However, in all cases the ranges of values were much reduced for the lower theta value (1.045). For instance, for GPP, the highest value obtained using a theta of 1.045 was around 4 O_2_ m^-2^ day^-1^ with most of the values below 3 O_2_ m^-2^ day^-1^, while for the higher theta value (1.1085), the highest value was twice as high (around 8 O_2_ m^-2^ day^-1^). Similar trends in low ranges for the lower theta value were observed for ER, GPP:ER and NEP. Moreover, the range in ER was very low (0.2-0.5 O_2_ m^-2^ day^-1^) (Figure S7b). This lack of variation in ER, which is very sensitive to temperature variation, to a low theta value (1.045) gave more credence to our use of a higher theta value (1.1085). Moreover, the higher theta value enabled us to successfully model more days, which enabled us to more effectively evaluate the effect of dung treatment on ecosystem metabolism in our mesocosms.

**Figure S7**. Performance of different values of theta in modeling metabolism in our experimental mesocosms. A higher value of theta (1.1085, upper panel) performed better for most streams when compared with a common literature value of 1.045 (lower panel). a and b are model fits for day 1, and c and d are model fits for day 10 in the hippo dung treatment (100 % hippo dung). The black bold line is for measured dissolved oxygen concentration (mg/L) while the red line is for the modeled dissolved oxygen concentration. The red dotted line is measured temperature and the blue dotted line is light intensity. The green dotted line is for oxygen saturation. Modeling for each day was performed from mid-night (0 minutes, 24:00 hrs) to mid-night, 1440 minutes, 23:59 hrs).

**Figure S8**. Model outputs using a theta value of 1.1085. Weekly measures of flume-scale gross primary production (GPP), (a) flume-scale ecosystem respiration (ER; b), GPP:ER (c) and net ecosystem production (NEP; d) using a theta value of 1.1085. The dotted line indicate NEP = 0, and most of the mesocosms were net heterotrophic on until day 7 and then switched.

**Figure S9**. Model outputs using a theta value of 1.045. Weekly measures of flume-scale gross primary production (GPP), (a) flume-scale ecosystem respiration (ER; b), GPP:ER (c) and net ecosystem production (NEP; d) using a theta value of 1.045. The dotted line indicate NEP = 0, and most of the mesocosms were net heterotrophic on until day 7 and then switched.

1. **Electronic supplementary material S6: Weekly measures of Ecosystem Metabolism**

To investigate weekly changes in ecosystem metabolism (GPP, ER, GPP/ER and net ecosystem production [NEP]), weekly means (1 value per stream per week, total 6 weeks) were used. Significant differences among dung treatments were tested using generalized additive mixed models [GAMMs, 69] after residuals in GLMM displayed non-linear responses to dung treatment. GAMM models included dung treatment as a fixed effect, and block and stream as random effects. Models were fitted using the the mgcv-package [70] in the R platform [71].

**Table S5**: Summary of generalized additive mixed modeling (GAMM) analyses to determine the effect of dung treatment on ecosystem metabolism - gross primary production (GPP, mg O_2_ m^-2^ day^-1^), ecosystem respiration (ER, ER, mg O_2_ m^-2^ day^-1^), GPP:ER and net ecosystem production (NEP, mg O_2_ m^-2^ day^-1^), which displayed nonlinear responses to dung treatments.

|  | **Measures of ecosystem metabolism** | | | |
| --- | --- | --- | --- | --- |
| **Variables** | **GPP** | **ER** | **GPP:ER** | **NEP** |
| Intercept (estimate(SE); t value | 4.05(0.36); 11.10*** | 0.77(0.04); 20.90*** | 4.50(0.36); 12.59*** | 3.41(0.34); 10.08*** |
| Dung Treatment (estimate(SE); t value | -0.07(0.01); -4.85*** | -0.01(<0.01); -5.17*** | -0.07(0.01); -4.67*** | -0.07(0.01); -4.67*** |
| Dung Treatment x Time (estimate(SE); t value | 0.01(<0.01); -4.81*** | <0.01(<0.01); -7.65*** | 0.01(<0.01); -4.65*** | 0.01(<0.01); -4.29*** |
| Block (EDF(F)) | <0.01 (0) | 0.81(1.81) | <0.01 (0) | <0.01 (0) |
| Adj. R2 | 0.41 | 0.40 | 0.44 | 0.44 |
| Explained deviance (%) | 48.7 | 43.8 | 51.7 | 51.7 |

SE= standard error; EDF = estimated degrees of freedom; *F* = ANOVA *F*-test value between the fitted and a null model. Significance: *P < 0.05, **P < 0.01, ***P < 0.001

**References**

[1] Edwards, P. 1991 Seasonal variation in the dung of African grazing mammals, and its consequences for coprophagous insects. *Functional Ecology*, 617-628.

[2] Sitters, J., Maechler, M.J., Edwards, P.J., Suter, W. & Olde Venterink, H. 2014 Interactions between C: N: P stoichiometry and soil macrofauna control dung decomposition of savanna herbivores. *Functional Ecology* **28**, 776-786.

[3] Codron, D., LEE‐THORP, J.A., Sponheimer, M., Codron, J., De Ruiter, D. & Brink, J.S. 2007 Significance of diet type and diet quality for ecological diversity of African ungulates. *Journal of Animal Ecology* **76**, 526-537.

[4] Sileshi, G.W., Nhamo, N., Mafongoya, P.L. & Tanimu, J. 2017 Stoichiometry of animal manure and implications for nutrient cycling and agriculture in sub-Saharan Africa. *Nutrient cycling in agroecosystems* **107**, 91-105.

[5] Subalusky, A.L., Dutton, C.L., Rosi-Marshall, E.J. & Post, D.M. 2015 The hippopotamus conveyor belt: vectors of carbon and nutrients from terrestrial grasslands to aquatic systems in sub-Saharan Africa. *Freshwater Biology* **60**, 512-525. (doi:10.1111/fwb.12474).

[6] Cerling, T.E., Harris, J.M. & Passey, B.H. 2003 Diets of East African Bovidae based on stable isotope analysis. *Journal of Mammalogy* **84**, 456-470.

[7] Kingdon, J. & Largen, M. 1997 The kingdom field guide to African mammals. *Zoological Journal of the Linnean Society* **120**, 479.

[8] Elliott, R. & Fokkema, K. 1961 Herbage consumption studies on beef cattle. 1. Intakestudies on Afrikander and Mashonacows onveldgrazing-1958/9. *Rhodesia Agricultural Journal* **58**, 49-57.

[9] Oyenuga, V. & Olubajo, F. 1975 Pasture productivity in Nigeria: II. Voluntary intake and herbage digestibility. *The Journal of Agricultural Science* **85**, 337-343.

[10] Clauss, M., Schwarm, A., Ortmann, S., Streich, W.J. & Hummel, J. 2007 A case of non-scaling in mammalian physiology? Body size, digestive capacity, food intake, and ingesta passage in mammalian herbivores. *Comparative Biochemistry and Physiology Part A: Molecular & Integrative Physiology* **148**, 249-265.

[11] Müller, D.W., Caton, J., Codron, D., Schwarm, A., Lentle, R., Streich, W.J., Hummel, J. & Clauss, M. 2011 Phylogenetic constraints on digesta separation: variation in fluid throughput in the digestive tract in mammalian herbivores. *Comparative Biochemistry and Physiology Part A: Molecular & Integrative Physiology* **160**, 207-220.

[12] Lamprey, R.H. & Reid, R.S. 2004 Expansion of human settlement in Kenya's Maasai Mara: what future for pastoralism and wildlife? *Journal of Biogeography* **31**, 997-1032.

[13] Hoffman, C.M. 2007 Geospatial mapping and analysis of water availability-demand-use within the Mara River Basin. *Florida International University, Miami*.

[14] Zhu, Y., Merbold, L., Pelster, D., Diaz‐Pines, E., Wanyama, G.N. & Butterbach‐Bahl, K. 2018 Effect of dung quantity and quality on greenhouse gas fluxes from tropical pastures in Kenya. *Global Biogeochemical Cycles* **32**, 1589-1604.

[15] Bond, T.A., Sear, D. & Edwards, M. 2012 Temperature-driven river utilisation and preferential defecation by cattle in an English chalk stream. *Livestock Science* **146**, 59-66.

[16] Clauss, M., Schwarm, A., Ortmann, S., Alber, D., Flach, E., Kühne, R., Hummel, J., Streich, W.J. & Hofer, H. 2004 Intake, ingesta retention, particle size distribution and digestibility in the hippopotamidae. *Comparative Biochemistry and Physiology Part A: Molecular & Integrative Physiology* **139**, 449-459.

[17] Reid, R.S., Rainy, M., Ogutu, J., Kruska, R., Kimani, K., Nyabenge, M., McCartney, M., Kshatriya, M., Worden, J. & Ng’ang’a, L. 2003 People, wildlife and livestock in the Mara ecosystem: The Mara count 2002. *International Livestock Research Institute, Nairobi, Kenya*.

[18] Kanga, E.M., Ogutu, J.O., Olff, H. & Santema, P. 2011 Population trend and distribution of the Vulnerable common hippopotamus Hippopotamus amphibius in the Mara Region of Kenya. *Oryx* **45**, 20-27.

[19] Ogutu, J.O., Piepho, H.-P., Said, M.Y., Ojwang, G.O., Njino, L.W., Kifugo, S.C. & Wargute, P.W. 2016 Extreme Wildlife Declines and Concurrent Increase in Livestock Numbers in Kenya: What Are the Causes? *PLOS ONE* **11**, e0163249. (doi:10.1371/journal.pone.0163249).

[20] Dijkslag, M., Elling-Staats, M., Yen, Y., Marchal, L. & Kwakkel, R. 2019 The effects of coarse and wet feeding on performance parameters, gastrointestinal tract and tibia traits, and digesta phytase activity in egg-type pullets, either fed a low or moderate phosphorus diet. *Poultry science* **98**, 4729-4744.

[21] Fritz, J., Hummel, J., Kienzle, E., Arnold, C., Nunn, C. & Clauss, M. 2009 Comparative chewing efficiency in mammalian herbivores. *Oikos* **118**, 1623-1632. (doi:doi:10.1111/j.1600-0706.2009.17807.x).

[22] Thomas, S. & Campling, R.C. 1977 Comparisons of some factors affecting digestibility in sheep and cows. *Grass and Forage Science* **32**, 33-41. (doi:doi:10.1111/j.1365-2494.1977.tb01409.x).

[23] Subalusky, A.L., Dutton, C.L., Njoroge, L., Rosi, E.J. & Post, D.M. 2018 Organic matter and nutrient inputs from large wildlife influence ecosystem function in the Mara River, Africa. *Ecology* **99**, 2558-2574.

[24] APHA. 1998 *Standard Methods for the Examination of Water and Wastewater*. Washington, D.C., APHA-AWWA-WEF.

[25] Coble, P.G. 2007 Marine optical biogeochemistry: the chemistry of ocean color. *Chemical reviews* **107**, 402-418.

[26] Ishii, S.K. & Boyer, T.H. 2012 Behavior of reoccurring PARAFAC components in fluorescent dissolved organic matter in natural and engineered systems: a critical review. *Environmental science & technology* **46**, 2006-2017.

[27] Fellman, J.B., Hood, E. & Spencer, R.G. 2010 Fluorescence spectroscopy opens new windows into dissolved organic matter dynamics in freshwater ecosystems: A review. *Limnol Oceanogr* **55**, 2452-2462.

[28] Masese, F.O., Salcedo-Borda, J.S., Gettel, G.M., Irvine, K. & McClain, M.E. 2017 Influence of catchment land use and seasonality on dissolved organic matter composition and ecosystem metabolism in headwater streams of a Kenyan river. *Biogeochemistry* **132**, 1-22.

[29] Mwanake, R., Gettel, G., Aho, K., Namwaya, D., Masese, F., Butterbach‐Bahl, K. & Raymond, P. 2019 Land use, not stream order, controls N2O concentration and flux in the upper Mara River basin, Kenya. *Journal of Geophysical Research: Biogeosciences*.

[30] Jaffé, R., McKnight, D., Maie, N., Cory, R., McDowell, W.H. & Campbell, J.L. 2008 Spatial and temporal variations in DOM composition in ecosystems: The importance of long-term monitoring of optical properties. *Journal of Geophysical Research: Biogeosciences* **113**. (doi:doi:10.1029/2008JG000683).

[31] Green, S.A. & Blough, N.V. 1994 Optical absorption and fluorescence properties of chromophoric dissolved organic matter in natural waters. *Limnol Oceanogr* **39**, 1903-1916. (doi:doi:10.4319/lo.1994.39.8.1903).

[32] Helms, J.R., Stubbins, A., Ritchie, J.D., Minor, E.C., Kieber, D.J. & Mopper, K. 2008 Absorption spectral slopes and slope ratios as indicators of molecular weight, source, and photobleaching of chromophoric dissolved organic matter. *Limnol Oceanogr* **53**, 955-969.

[33] Weishaar, J.L., Aiken, G.R., Bergamaschi, B.A., Fram, M.S., Fujii, R. & Mopper, K. 2003 Evaluation of Specific Ultraviolet Absorbance as an Indicator of the Chemical Composition and Reactivity of Dissolved Organic Carbon. *Environmental Science & Technology* **37**, 4702-4708. (doi:10.1021/es030360x).

[34] McKnight, D.M., Boyer, E.W., Westerhoff, P.K., Doran, P.T., Kulbe, T. & Andersen, D.T. 2001 Spectrofluorometric characterization of dissolved organic matter for indication of precursor organic material and aromaticity. *Limnol Oceanogr* **46**, 38-48.

[35] Parlanti, E., Wörz, K., Geoffroy, L. & Lamotte, M. 2000 Dissolved organic matter fluorescence spectroscopy as a tool to estimate biological activity in a coastal zone submitted to anthropogenic inputs. *Organic Geochemistry* **31**, 1765-1781. (doi:<https://doi.org/10.1016/S0146-6380(00)00124-8>).

[36] Wilson, H.F. & Xenopoulos, M.A. 2009 Effects of agricultural land use on the composition of fluvial dissolved organic matter. *Nature Geoscience* **2**, 37.

[37] Ohno, T. 2002 Fluorescence inner-filtering correction for determining the humification index of dissolved organic matter. *Environmental science & technology* **36**, 742-746.

[38] Huguet, A., Vacher, L., Relexans, S., Saubusse, S., Froidefond, J.-M. & Parlanti, E. 2009 Properties of fluorescent dissolved organic matter in the Gironde Estuary. *Organic Geochemistry* **40**, 706-719.

[39] Zsolnay, A., Baigar, E., Jimenez, M., Steinweg, B. & Saccomandi, F. 1999 Differentiating with fluorescence spectroscopy the sources of dissolved organic matter in soils subjected to drying. *Chemosphere* **38**, 45-50.

[40] Stedmon, C.A. & Bro, R. 2008 Characterizing dissolved organic matter fluorescence with parallel factor analysis: a tutorial. *Limnology and Oceanography: Methods* **6**, 572-579. (doi:doi:10.4319/lom.2008.6.572b).

[41] Huber, S.A., Balz, A., Abert, M. & Pronk, W. 2011 Characterisation of aquatic humic and non-humic matter with size-exclusion chromatography–organic carbon detection–organic nitrogen detection (LC-OCD-OND). *Water research* **45**, 879-885.

[42] Murphy, K.R., Stedmon, C.A., Wenig, P. & Bro, R. 2014 OpenFluor–an online spectral library of auto-fluorescence by organic compounds in the environment. *Analytical Methods* **6**, 658-661.

[43] Coble, P.G. 1996 Characterization of marine and terrestrial DOM in seawater using excitation-emission matrix spectroscopy. *Marine chemistry* **51**, 325-346.

[44] Stedmon, C.A. & Markager, S. 2005 Resolving the variability in dissolved organic matter fluorescence in a temperate estuary and its catchment using PARAFAC analysis. *Limnol. Oceanogr.* **50**, 686-697.

[45] Cory, R.M. & McKnight, D.M. 2005 Fluorescence spectroscopy reveals ubiquitous presence of oxidized and reduced quinones in dissolved organic matter. *Environmental science & technology* **39**, 8142-8149.

[46] Wu, F., Evans, R. & Dillon, P. 2003 Separation and characterization of NOM by high-performance liquid chromatography and on-line three-dimensional excitation emission matrix fluorescence detection. *Environmental science & technology* **37**, 3687-3693.

[47] Coble, P.G., Green, S.A., Blough, N.V. & Gagosian, R.B. 1990 Characterization of dissolved organic matter in the Black Sea by fluorescence spectroscopy. *Nature* **348**, 432.

[48] Coble, P.G., Del Castillo, C.E. & Avril, B. 1998 Distribution and optical properties of CDOM in the Arabian Sea during the 1995 Southwest Monsoon. *Deep Sea Research Part II: Topical Studies in Oceanography* **45**, 2195-2223.

[49] Stedmon, C.A. & Markager, S. 2005 Tracing the production and degradation of autochthonous fractions of dissolved organic matter by fluorescence analysis. *Limnol. Oceanogr.* **50**, 1415-1426.

[50] Murphy, K.R., Stedmon, C.A., Waite, T.D. & Ruiz, G.M. 2008 Distinguishing between terrestrial and autochthonous organic matter sources in marine environments using fluorescence spectroscopy. *Mar. Chem.* **108**, 40-58. (doi:Doi 10.1016/J.Marchem.2007.10.003).

[51] Murphy, K.M., K. R., Hambly, A., Singh, S., Henderson, R.K., Baker, A., Stuetz, R. & Khan, S.J. 2011 Organic Matter Fluorescence in Municipal Water Recycling Schemes: Toward a Unified PARAFAC Model. *Environ. Sci. Technol.* **45**, 2909-2916. (doi:10.1021/es103015e).

[52] Stedmon, C.A., Markager, S. & Bro, R. 2003 Tracing dissolved organic matter in aquatic environments using a new approach to fluorescence spectroscopy. *Mar. Chem.* **82**, 239-254. (doi:Doi 10.1016/S0304-4203(03)00072-0).

[53] Cory, R.M. & Kaplan, L.A. 2012 Biological lability of streamwater fluorescent dissolved organic matter. *Limnol. Oceanogr.* **57**, 1347-1360.

[54] Fuss, T., Behounek, B., Ulseth, A.J. & Singer, G.A. 2017 Land use controls stream ecosystem metabolism by shifting dissolved organic matter and nutrient regimes. *Freshwater Biol* **62**, 582-599. (doi:10.1111/fwb.12887).

[55] Hotchkiss, E.R. & Hall, R.O. 2014 High rates of daytime respiration in three streams: Use of delta O-18(O2) and O-2 to model diel ecosystem metabolism. *Limnol Oceanogr* **59**, 798-810. (doi:10.4319/lo.2014.59.3.0798).

[56] Van de Bogert, M.C., Carpenter, S.R., Cole, J.J. & Pace, M.L. 2007 Assessing pelagic and benthic metabolism using free water measurements. *Limnol Oceanogr-Meth* **5**, 145-155. (doi:DOI 10.4319/lom.2007.5.145).

[57] Marzolf, E.R., Mulholland, P.J. & Steinman, A.D. 1998 Reply: Improvements to the diurnal upstream-downstream dissolved oxygen change technique for determining whole-stream metabolism in small streams. *Can J Fish Aquat Sci* **55**, 1786-1787.

[58] Odum, H.T. 1956 Primary Production in Flowing Waters. *Limnol Oceanogr* **1**, 102-117. (doi:DOI 10.4319/lo.1956.1.2.0102).

[59] Kirschbaum, M.U. 1995 The temperature dependence of soil organic matter decomposition, and the effect of global warming on soil organic C storage. *Soil Biology and biochemistry* **27**, 753-760.

[60] Parkhill, K.L. & Gulliver, J.S. 1999 Modeling the effect of light on whole-stream respiration. *Ecological Modelling* **117**, 333-342.

[61] Demars, B.O., Thompson, J. & Manson, J.R. 2015 Stream metabolism and the open diel oxygen method: Principles, practice, and perspectives. *Limnology and Oceanography: Methods* **13**, 356-374.

[62] Benson, B.B. & Krause Jr, D. 1984 The concentration and isotopic fractionation of oxygen dissolved in freshwater and seawater in equilibrium with the atmosphere 1. *Limnol Oceanogr* **29**, 620-632.

[63] Raymond, P.A., Zappa, C.J., Butman, D., Bott, T.L., Potter, J., Mulholland, P., Laursen, A.E., McDowell, W.H. & Newbold, D. 2012 Scaling the gas transfer velocity and hydraulic geometry in streams and small rivers. *Limnology and Oceanography: Fluids and Environments* **2**, 41-53.

[64] Elmore, H. 1961 Effects of water temperature on stream sanitation. *J. Sanitary Eng.* **87**, 59-71.

[65] Bott, T.L. 1996 Primary productivity and community respiration. *Methods in stream ecology*, 533-556.

[66] Perkins, D.M., Yvon-Durocher, G., Demars, B.O.L., Reiss, J., Pichler, D.E., Friberg, N., Trimmer, M. & Woodward, G. 2012 Consistent temperature dependence of respiration across ecosystems contrasting in thermal history. *Global Change Biol* **18**, 1300-1311. (doi:doi:10.1111/j.1365-2486.2011.02597.x).

[67] Sand-Jensen, K., Pedersen, N.L. & Sondergaard, M. 2007 Bacterial metabolism in small temperate streams under contemporary and future climates. *Freshwater Biol* **52**, 2340-2353. (doi:doi:10.1111/j.1365-2427.2007.01852.x).

[68] Riley, A.J. & Dodds, W.K. 2013 Whole-stream metabolism: strategies for measuring and modeling diel trends of dissolved oxygen. *Freshwater Science* **32**, 56-69. (doi:10.1899/12-058.1).

[69] Zuur, A., Ieno, E.N. & Smith, G.M. 2007 *Analyzing ecological data*, Springer Science & Business Media.

[70] Wood, S. & Wood, M.S. 2015 Package ‘mgcv’. *R package version*, 1-7.

[71] R Core Team. 2017 R: A language and environment for statistical computing. (3.3.1 ed. Vienna, Austria, R Foundation for Statistical Computing.
